# Assessing perceptions of resilience: The understanding from network analysis

**DOI:** 10.3389/fpubh.2023.1017871

**Published:** 2023-01-20

**Authors:** Rong Liu, Wenjie Duan

**Affiliations:** ^1^Student Counseling and Mental Health Center, East China University of Science and Technology, Shanghai, China; ^2^Social and Public Administration School, East China University of Science and Technology, Shanghai, China

**Keywords:** resilience, multi-systems, psychometric, factor structure, network analysis

## Abstract

**Introduction:**

Previous studies have yet to reach a consensus on the construct of resilience perception, and how to enhance the effect of resilience intervention remains an urgent issue. In this consideration, this study examines the fundamental construct of resilience. It provides insight into the critical prevention goal for resilience intervention by utilizing the latest methods of psychological network analysis.

**Methods:**

The sample is the graduate students enrolled in September 2021. Participants completed (1) the Connor-Davidson Resilience Scale, (2) the University of Washington Resilience Scale-8 Item, (3) the Brief Resilience Scale, and (4) the Resilience Scale for Adults, each representing different orientations of resilience.

**Results:**

The network analysis grants greater clarity to the resilience perception as a dynamic system that interacts between an individual's tendency to intrinsic capacity and response to external resources. This study has shown that a positive perception of external social resources is the most important for individuals' resilience cognition; the effect of resilience intervention can be achieved more quickly by changing the individual's sense of hope.

**Discussion:**

Based on the results, a psychometric instrument that integrates different orientations of resilience concepts and is based on time-varying needs to be developed.

## 1. Introduction

The idea of resilience has become prevalent in international development (1) due to mounting global risks (2–4). The most typical study of resilience is the traumatic resilience research (5–7). Recently, researchers have realized that shifting from the focus on the traditional methods of psychological disorder treatment, such as posttraumatic stress disorder, to the maintenance of stress-related mental health is a promising strategy, which helps to narrow the prevention gap (8–11). Bonanno et al. (12) recommended that individual resilience has an important contribution to the prevention of anxiety and depression. Therefore, resilience is conceptualized as the maintenance or quick recovery of a healthy mental state during and after adversity (5, 11).

This study focuses on individual resilience perception assessment. The concept of resilience has been widely used in interdisciplinary research to deal with interference and change, involving psychological, social, ecological, economic, neurological, and biological categories (2, 13). The resilience science of dynamic multisystem emphasizes the interaction between system and individual (14). Liu et al. (15) presented a multisystem model of resilience (MSMR) to support the hypotheses of three systems. In this model, the innermost system includes health and health-related internal resources within the individual. The intermediate system reflects the individual's tendency and response to life and the external environment as a dynamic process bridging internal and external systems. The outermost system is the social and ecological resources of resilience that act as the external system. The perception of individual resilience is similar to the intermediate system, so we assume that the resilience structure includes the tendency to internal characteristics and the response to external resources. However, Windle et al. (16) noted that contemporary resilience questionnaires contribute minimally to predicting an individual's positive adaptation after adversity. Focusing on different facets of the resilience construct may be the cause of inconsistent resilience estimates (17). For example, the meta-analysis of resilience intervention measured by Liu et al. (18) showed that although these studies have achieved remarkable statistical results, the overall effects are limited. Therefore, how to enhance the effect of resilience intervention remains an urgent issue (19).

The critical to resilience as disease prevention may be the consistency in resilience estimates. The construct of resilience varies in its emphasis on capacity, process, outcome, and protective resources (20). Therefore, the existing scales with higher scores of individual resilience are selected to verify the most important structure of resilience and the relationship between them to better improve the effectiveness of resilience intervention. Capacity-oriented resilience is defined as a fixed individual characteristic, which helps to identify resilient qualities that facilitate recovery from adversity (21–24). The Connor–Davidson Resilience Scale (CD-RISC) is the representative psychometric sound scale for capacity-oriented resilience (25). It has shown good validity and reliability in America (25), Africa (26), and China (27). Although satisfactory psychometric properties were reported, the construct validity of resilience has always been a controversial issue (28). Process-oriented resilience focuses on the specific reaction and response process when an individual is threatened (29). This approach to resilience attempts to answer the question of “how resilient qualities are acquired” (21, 30). The University of Washington Resilience Scale-8 Item (UWRS-8) is a more recent questionnaire calibrated to modern psychometric methods with scores on a T-metric (31). The UWRS-8 has been verified by validity and reliability in American (31) and international samples (51.4% Europe, 30.4% America, and 18.2% others) (32) but is lacking for Chinese samples. Outcome-oriented resilience concerns whether individuals have recovered from adversity and exhibited positive adaptation (33). In other words, it primarily focuses on the binary question of whether adversity has been overcome (20). The Brief Resilience Scale (BRS) is the representative psychometrically sound scale for outcome-oriented resilience (34). It has been verified by good validity and reliability in America (34) and China (35), and psychometric properties were reported. Unlike the orientations discussed above, protective resources-oriented resilience is viewed from outside an individual's interpersonal and social environment (14). It highlights an individual's interdependence with the various systems in his or her life (14). The Resilience Scale for Adults (RSA) is the representative psychometric sound scale for protective resources-oriented resilience (36). It has been verified by validity and reliability in Norway (36), Africa (37), and China (38). Although satisfactory psychometric properties were reported, the construct validity of resilience has always been a controversial issue.

Studies have increasingly shown that traditional approaches are limited in verifying the multifactor resilience scale (39). The current studies cannot fit the prediction of resilience because they are limited by traditional assumptions, that is, resilience is not a dynamic adaptation system but is measured as a stable trait (11). Psychopathological network analysis provides a new method for explaining the complex dynamics of mental health (40). Contrary to the traditional accounts that view an episode of disorder as a potential and unobservable disease entity, network analysis considers an episode as the causal interactions between its symptom elements, directly reflecting the psychological process of individuals in nature (41–46). For example, Bringmann et al. (47) uses network analysis to dynamically assess the depressive symptoms of patients during 14 weeks of treatment, revealing more clearly the direct and indirect connections between symptoms through time-dependent patterns. Network analysis can identify the core projects that make it possible to develop more effective treatment strategies by examining the centrality of symptoms and community structures.

For the present study, the long-term adverse risks to people's mental health, such as anxiety and depression, have increased remarkably with the spread of the COVID-19 pandemic, making mental illness a more serious influence on individual and even public health (48, 49). In accordance with the Report on National Mental Health Development in China (2019–2020), the average level of anxiety among young adults aged 18–34 is remarkably higher than in other age groups. Evans et al. (50) pointed out that graduate students are more than six times more likely to experience depression and anxiety than ordinary people. In 2019, Nature magazine surveyed 6,300 early professional researchers in various scientific fields around the world, and more than 36% of the respondents sought help due to anxiety and depression caused by overwork, the economy, imbalance, and future uncertainty. It also showed another aspect of stress, namely, greater personal satisfaction and resilience in this context (51). Thus, we choose this group to investigate the structure and important nodes of resilience to better understand the perceptions of individual resilience. First, we will measure the resilience of the four networks. We will then evaluate the complex network that integrates the four different resilience measurements (1) to investigate whether the construct compared with the network is consistent with the hypothesis, (2) to explain the resilience structure and the relationship between them, and (3) to find the key prevention goal for resilience intervention.

## 2. Method

### 2.1. Participants and procedure

This study selected Chinese graduate samples. In accordance with 3.77 million graduate students enrolled in 31 provinces and municipalities throughout the country in 2021, the proportion of students enrolled in the eastern, central, and western regions of China is 51: 30: 19 (52). This study used stratified sampling to extract 1,275 people from the eastern region, including Beijing, Shanghai, Guangdong, and other 11 provinces and municipalities; 750 people in the central region, including eight provinces, such as Shanxi, Jilin, and Heilongjiang; 475 people in the western region, including 12 provinces and municipalities, such as Inner Mongolia, Guangxi, and Chongqing, and conducted an online questionnaire survey on the research samples from September to October 2021. Data sieving: insufficient effort response and response time analysis were used to avoid statistically and significantly biased estimates and invalid inferences. Participants included 1,896 graduate students (male = 956, female = 940; mean age = 22.74, SD = 1.215, range = 20–27). Detailed demographic information can be found in Table 1. All participants completed the four scales: the CD-RISC, the UWRS-8, the BRS, and the RSA. All analyses were run on Jeffreys' Amazing Statistics Program. More information can be found in Love et al. (53). The Human Subjects Ethics Subcommittee has approved this study.

**Table 1:** Psychometric properties for resilience scales and subscales (*N* = 1,896).

	**Items**	**M**	**SD**	**Range**	**Cronbach's α**
Tenacity	13	28.27	5.088	8–40	0.90
Strength	8	29.89	4.278	8–40	0.76
Optimism	4	15.26	2.639	4–20	0.72
UWRS-8	8	30.78	4.863	8–40	0.90
BRS	13	21.08	3.435	7–30	0.71
Perception of self	6	30.94	5.257	8–42	0.75
Planned future	4	20.71	4.417	4–28	0.80
Structure style	4	20.93	3.752	10–28	0.55
Social competence	6	30.31	6.032	9–42	0.75
Family cohesion	6	32.95	6.123	6–42	0.79
Social resources	7	41.61	6.105	13–49	0.81

Tenacity, strength, and optimism are the three subscales of CD-RISC that represent the capacity-oriented resilience, UWRS-8 and BRS are unidimensional scales that separately represent the process-oriented and outcome-oriented resilience. Perception of self, planned future, structure style, social competence, family cohesion, and social resources are the six subscales of RSA that represent the protective resources of resilience.

### 2.2. Measures

#### 2.2.1. CD-RISC

The capacity of resilience was measured by the CD-RISC comprising 25 items. Each item was scored from 0 (“not true at all”) to 4 (“true nearly all of the time”). A higher aggregate score indicated greater resilience. A previous study demonstrated good internal reliabilities in the American population of graded prevention (Cronbach's α higher than 0.89) (25). The Chinese version demonstrated satisfactory consistency (Cronbach's α = 0.60–0.88) in the general population among different ages; however, the factor constructs were modified as “tenacity,” “strength,” and “optimism” (27). In this study, the scale has good internal reliability of the three-factor construct in university samples (Cronbach's α = 0.72–0.93).

#### 2.2.2. UWRS-8

The process of resilience was measured by the UWRS-8 comprising 8 items (31). Each item was scored from 1 (“not at all”) to 5 (“very much”). A higher aggregate score indicated greater resilience. A previous study demonstrated good internal reliability in the physically disabled and the general population in the USA (Cronbach's α higher than 0.8) (31). The Chinese version has not been tested. In this study, the scale has good internal reliability in university samples (Cronbach's α = 0.9).

#### 2.2.3. BRS

Resilience outcomes were measured by the BRS (34) comprising six items. Each item was scored from 1 (“strongly disagree”) to 5 (“strongly agree”)—the reverse coding items were 2, 4, and 6. A higher aggregate score indicated greater resilience. A previous study demonstrated good internal reliability and test-retest reliability in the patients and general population in the USA (Cronbach's α = 0.80–0.91, *r* = 0.69) (34). The Chinese sample demonstrated satisfactory consistency (Cronbach's α = 0.71) and good validity (35). In this study, the scale has good internal reliability in university samples (Cronbach's α = 0.708).

#### 2.2.4. RSA

The protective resources of resilience were measured by the RSA (36, 54) comprising 33 items that assessed five general resilience protective resources. Each item was scored from 1 (“not true at all”) to 5 (“true nearly all of the time”)—the reverse coding items included 16 items. A higher aggregate score indicated greater resilience. A previous study demonstrated good internal reliability in patients with psychiatric diagnoses in Norway (Cronbach's α higher than 0.8) (54). The Chinese version showed satisfactory consistency reliability (Cronbach's α = 0.76 to 0.87) and good validity (38). In this study, the scale has good internal reliability of the three-factor construct in university samples (Cronbach's α = 0.55–0.81).

The network consists of nodes and edges, where the nodes indicate the research object, and the edges indicate the connections between nodes, similar to the neurons in the neural network. The strength centrality is the number of edges connecting it to other nodes in the network, which represents the most critical node. The betweenness centrality is the number of times that a node appears on the shortest path between two other nodes. If it is removed, then it will decrease the speed of network transmission. The closeness centrality is the average distance between it and all other nodes in the network, which can be transferred information to other nodes in the network more quickly (41, 55).

## 3. Results

### 3.1. Descriptive and correlation statistics

Mean, standard deviation, range, and Cronbach's α values are provided in Table 1. In this study, the four scales have shown good internal reliability (CD-RISC = 0.72–0.90; UWRS-8 = 0.90; BRS = 0.71; RSA = 0.55–0.81). Tenacity, strength, and optimism are the three subscales of CD-RISC, and perception of self, planned future, structure style, social competence, family cohesion, and social resources are the six subscales of RSA. The BRS, the UWRS, and the subscales of CD-RISC and RSA have a high correlation with each other in Table 2.

**Table 2:** Bivariate correlations (Pearson's *r*) among resilience variables (*N* = 1,896).

	**1**	**2**	**3**	**4**	**5**	**6**	**7**	**8**	**9**	**10**	**11**
1. Tenacity	1										
2. Strength	0.870^**^	1									
3. Optimism	0.662^**^	0.706^**^	1								
4. UWRS-8	0.743^**^	0.732^**^	0.589^**^	1							
5. BRS	0.601^**^	0.587^**^	0.464^**^	0.631^**^	1						
6. Perception of self	0.605^**^	0.587^**^	0.528^**^	0.604^**^	0.627^**^	1					
7. Planned future	0.550^**^	0.525^**^	0.526^**^	0.539^**^	0.508^**^	0.671^**^	1				
8. Structures style	0.313^**^	0.342^**^	0.383^**^	0.351^**^	0.361^**^	0.463^**^	0.510^**^	1			
9. Social competence	0.478^**^	0.504^**^	0.481^**^	0.433^**^	0.410^**^	0.509^**^	0.458^**^	0.385^**^	1		
10. Family cohesion	0.297^**^	0.308^**^	0.307^**^	0.317^**^	0.309^**^	0.392^**^	0.406^**^	0.336^**^	0.426^**^	1	
11. Social resources	0.422^**^	0.426^**^	0.457^**^	0.415^**^	0.408^**^	0.513^**^	0.516^**^	0.417^**^	0.535^**^	0.599^**^	1
M	28.27	29.89	15.26	21.08	30.78	30.94	20.71	20.93	30.31	32.95	41.61
SD	5.088	4.278	2.639	3.435	4.863	5.257	4.417	3.752	6.032	6.123	6.105
Cronbach's α	0.901	0.764	0.720	0.708	0.900	0.753	0.799	0.554	0.753	0.787	0.808

^**^p < 0.01.

### 3.2. Network analysis of a single scale of resilience

**Figure 1:**
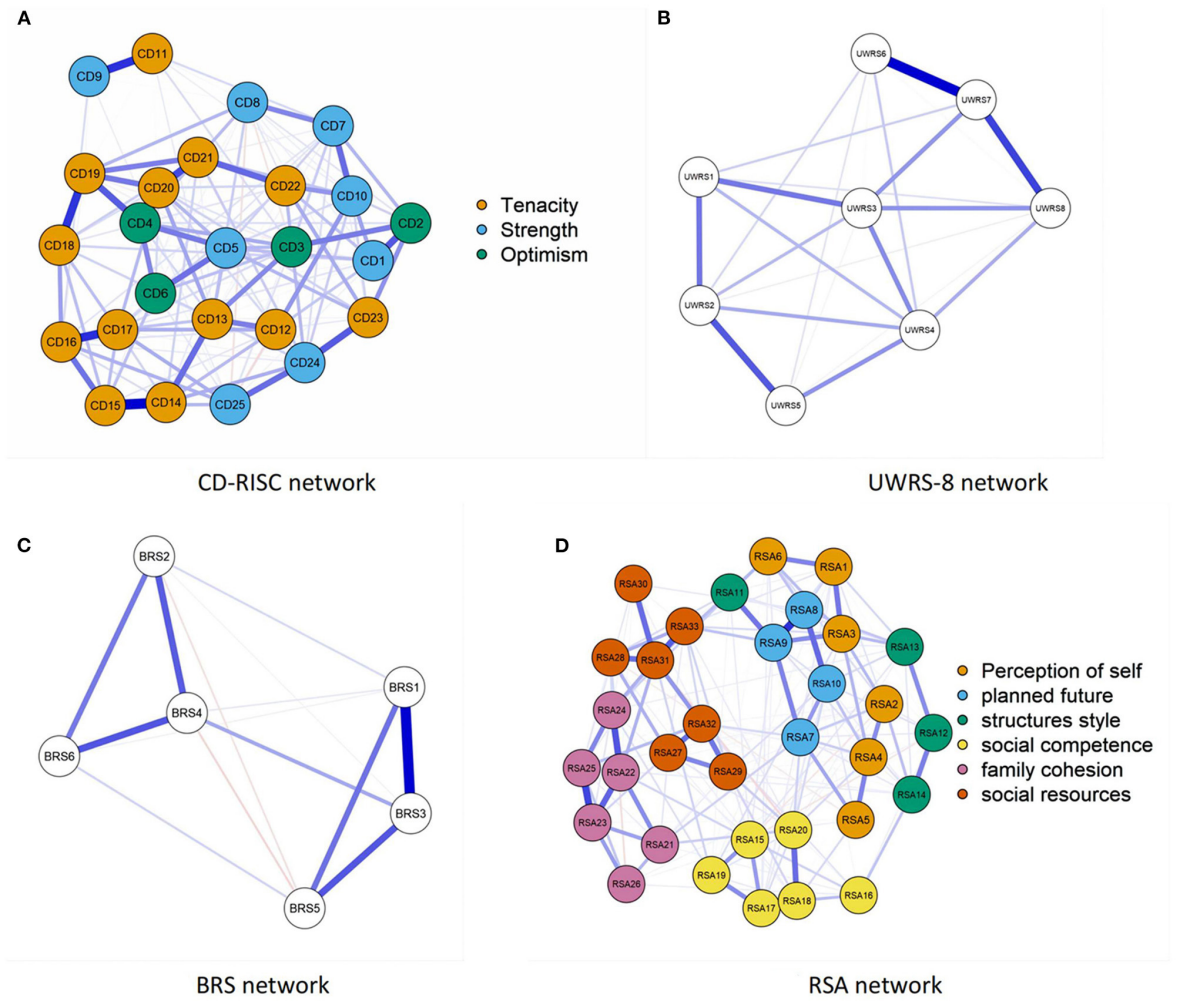
Structure of factors' network of resilience as measured by four single scales. **(A)** CD-RISC network. **(B)** UWRS-8 network. **(C)** BRS network. **(D)** RSA network.

**Figure 2:**
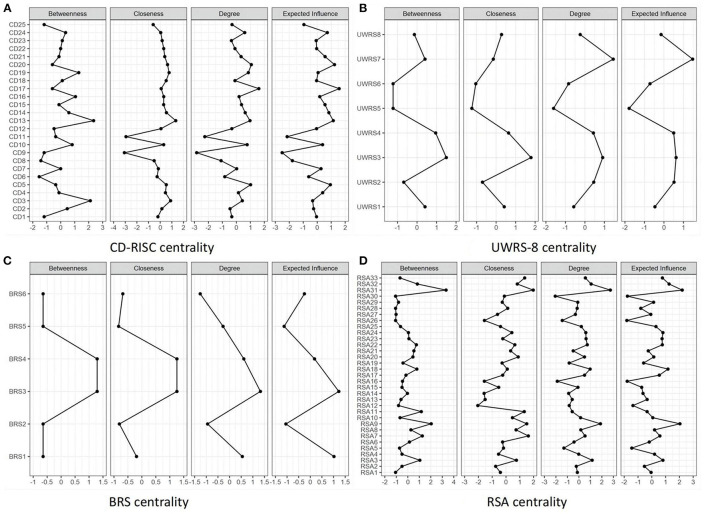
Centrality network of resilience as measured by four single scales. **(A)** CD-RISC centrality. **(B)** UWRS-8 centrality. **(C)** BRS centrality. **(D)** RSA centrality.

The network analysis structure of the four facets of resilience assessed using CD-RISC, UWRS-8, BRS, and RSA are shown in Figures 1A–D, respectively. The CD-RISC [CD1–CD25, (a)], the UWRS-8 [UWRS1–UWRS8, (b)], the BRS [BRS1–BRS6, (c)], and the RSA [RSA1–RSA33, (d)] show four different orientation resilience network analysis structures. Positive correlation between nodes is expressed by blue lines, negative correlation by red lines, and correlation intensity by edge thickness and brightness. The centrality of each scale of the resilience network can be found in Figures 2A–D. The (a) CD-RISC centrality, the (b) UWRS-8 centrality, the (c) BRS centrality, and the (d) RSA centrality show four different orientation resilience network centralities, including node strength, closeness, and betweenness.

For the CD-RISC network, the constructs of resilience representing the three-domain networks did not perform as expected. The highest node strength was CD17 (Think of self as a strong person), and the lowest was CD9 (Have to act on a hunch). CD13 (Past success gives confidence for a new challenge) had the highest node closeness and betweenness, node closeness was the lowest for CD9 (Have to act on a hunch), and node betweenness was the lowest for CD6 (See the humorous side of things).

For the UWRS-8 network, the highest node strength was UWRS7 (When something stressful happens, I keep going), and node betweenness and closeness were the highest for UWRS3 (When I experience a setback, I keep moving forward). By contrast, node strength, betweenness, and closeness were the lowest for UWRS5 (During stressful times, I am usually calm and relaxed).

For the BRS network, BRS3 (It does not take me long to recover from a stressful event) and BRS4 (It is hard for me to snap back when something bad happens) had the highest node strength, betweenness, and closeness, and BRS3 had the highest node strength. Contrarily, BRS6 (I tend to take a long time to get over setbacks in my life) had the lowest node strength, and node closeness was the lowest for BRS5 (I usually come through difficult times with little trouble).

For the RSA network, the resilience network showed that the constructs of resilience were unstable in the six-domain network, and the three factors were mixed. The node with the highest node strength, betweenness, and closeness was RSA31 (I get support from friends/family members), node strength and betweenness were the lowest for RSA30 (When a family member experiences a crisis/emergency I am informed right away), and node closeness was the lowest for RSA12 (When I start on new things/projects, I rarely plan, just get on with it).

### 3.3. Resilience construct network analysis of CD-RISC, UWRS-8, BRS, and RSA

**Figure 3:**
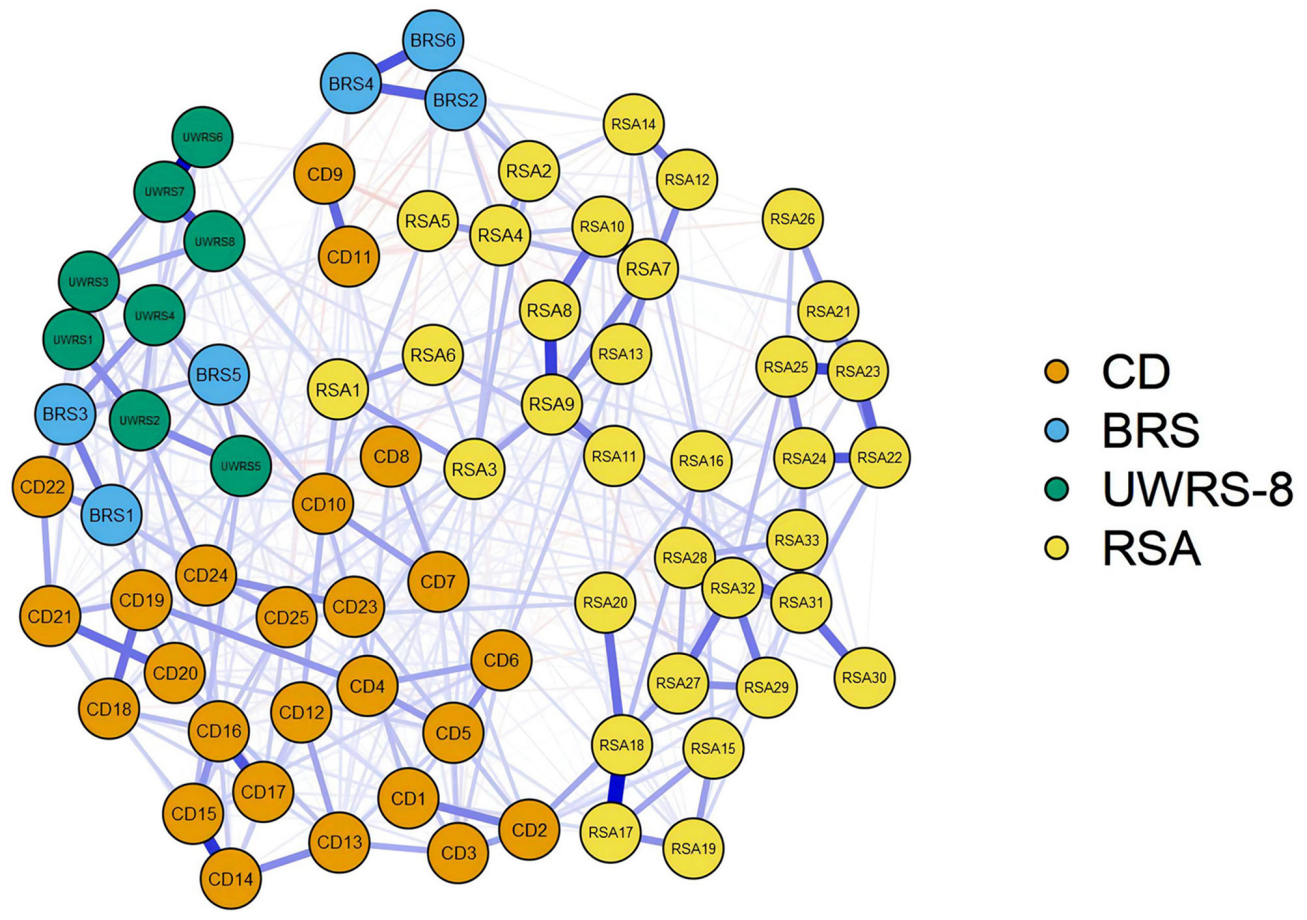
Resilience structure network analysis of CD-RISC, UWRS-8, BRS, and RSA.

**Figure 4:**
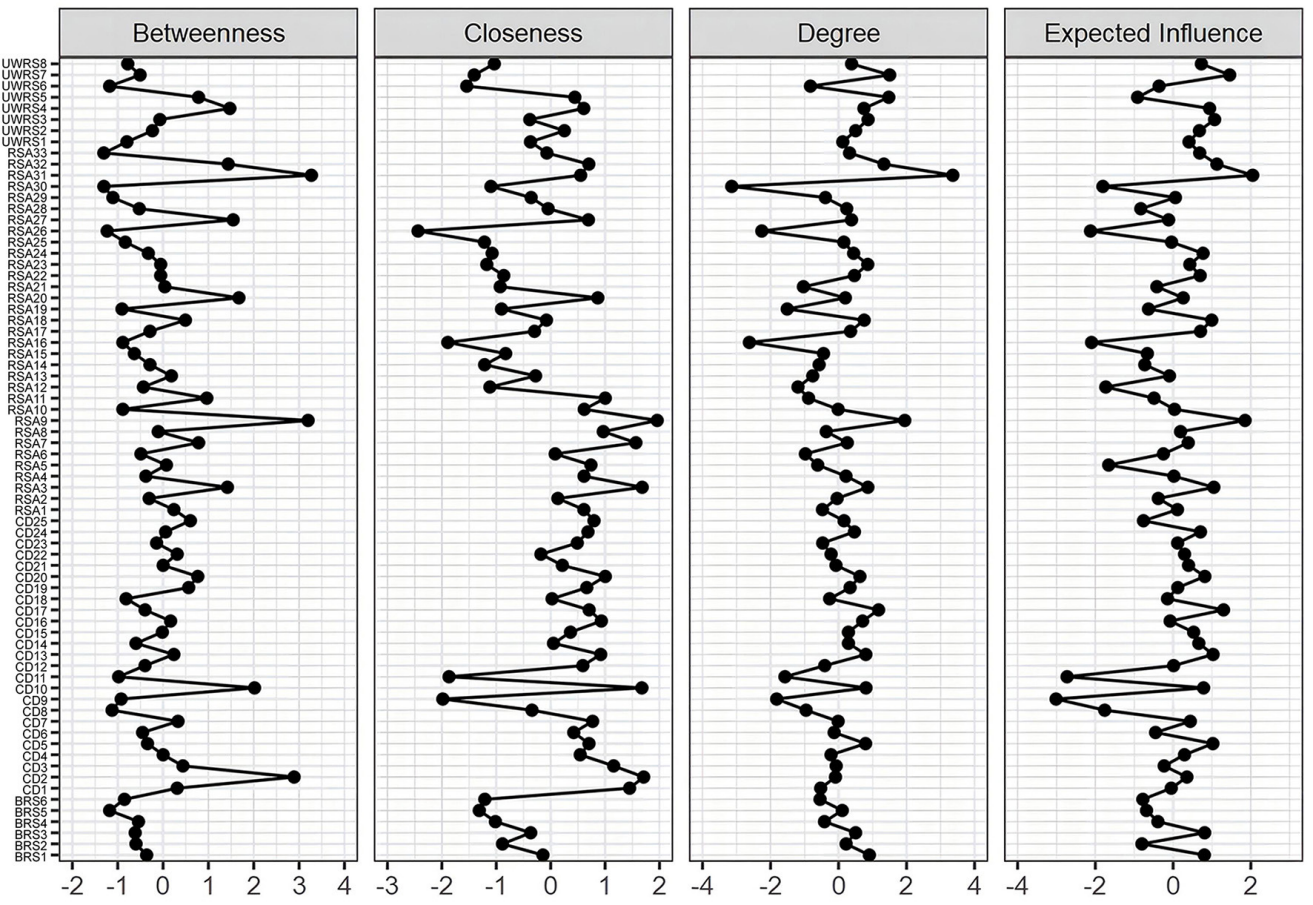
Centrality network analysis of CD-RISC, UWRS-8, BRS, and RSA.

The four combined network analysis structures with different orientations of resilience are shown in Figure 3 to investigate resilience as measured by the four scales of the CD-RISC, UWRS-8, BRS, and RSA, representing capacity, process, outcome, and protective resources of resilience, respectively The centrality of the four different orientation resilience combined network can be found in Figure 4.

For the combined network, the CD-RISC and RSA were connected and relatively independent, and the nodes of UWRS and BRS were confused with other constructs of resilience. The node strength was the highest for RSA31 (I get support from friends/family members) and RSA9 (I feel that my future looks very promising), and betweenness was the highest for RSA31 (I get support from friends/family members) and RSA9 (I feel that my future looks very promising). The node with the highest closeness was RSA9 (I feel that my future looks very promising) and RSA3 (Belief in myself gets me through difficult periods). Positive associations emerged between UWRS4 (Although I feel bad sometimes, I usually bounce right back) and CD10 (Can handle unpleasant feelings), UWRS2 (When something happens that makes me feel stressed, I usually calm down quickly) and CD24 (Under pressure, focus and think clearly), and CD2 (Close and secure relationships) and RSA27 (I can discuss personal issues with No one).

## 4. Discussion and applications to practice

This research aims to examine whether the structure of resilience is consistent with the hypothesis and find the key prevention goal from the network analysis. Compared with the limitations of the traditional approaches (56), the proposed method performed better in capturing the true construct of resilience. The network analysis is a breakthrough in methodology and development theory, which provides reliable and effective resilience measurement methods that enable us to achieve more accurate results in personalized treatment (57–59). This study demonstrated that the resilience structure of capacity and protection resources is obvious. A high correlation is observed between the four scales, so its capacity and protective resources of resilience are interrelated rather than heterogeneous. The results support the MSMR theoretical model that the structure of individual resilience perception is not capacity, process, outcome, and resource (15), but a state of mind that interaction between individual's tendency to intrinsic capacity and response to external resources (60). Resilience is an adaptive process of fluidity and interaction rather than an individual characteristic (57).

Protective resources-oriented resilience is considered a decisive factor in the resilience structure. The highest nodes of strength, closeness, and betweenness were found in the RSA, which represented resilience to external responses, including access to external support, services, and environmental resources (15). However, how to enhance the effect of resilience intervention remains an urgent issue. Kalisch et al. (61) proposed a unified theoretical framework for neuroscience research on general resilience mechanisms. The positive appraisal style is a key resilience mechanism through which all resilience factors converge and affect resilience. The highest node of strength is RSA31 (I get support from friends/family members). Studies have shown that individuals' positive perception of external social resources, such as the belief that they can get social support, is the most important for individuals' resilience cognition (62). The highest node of closeness is RSA9 (I feel that my future looks very promising) and RSA3 (Belief in myself gets me through difficult periods). Positive perceptions of future outcomes indicate that they will have positive experiences or potential negative situations will not occur, and that individuals think of their ability to cope with the aversive situation. When the two aspects change, individuals can adapt to the current obstacles more quickly (63, 64). Hope theory demonstrates that one's perception of the ways to overcome obstacles and the motivation to use these ways to achieve goals plays an important role in the adaptive response to obstacles (65).

However, this study has some limitations. First, this study only delved into the structure of resilience by collecting existing scales that abstract resilience into potential variables developed using traditional measurements. A new psychometric instrument is needed to depict resilience factors directly. For instance, the hybrid symptom-and-resilience factor models proposed by Kalisch et al. (60) directly introduce the resilience factors into the mental symptom network, deconstruct resilience into entities, and maintain individual mental health by weakening the interconnection of symptoms. The resilience network can better explain the dynamics of mental health maintenance in the process of stress exposure. Second, a tool that can identify the time-varying efficiency of resilience factors must be developed to study the dynamic characteristics of individual networks. Previous and current studies usually infer from the cross sectional analysis at the group level. In accordance with ecological fallacy theory, the pattern at the group level may be completely different from that at the individual level even if individuals are homogeneous (66, 67). Therefore, cross sectional analysis cannot capture the psychological process' variables and time-varying natural attributes (55). Previous studies showed that network analysis can better describe the process of time-varying external influence and internal interaction. For instance, the multilevel vector autoregressive time-series model has been able to evaluate how variables change with time in the same measurement window and predict each other at the previous and next point in time (55, 68–70). Thus, the new measurement can enable resilience intervention efficacy. Third, this study did not explore how variables external to the network itself affect network dynamics. The interaction between individuals and the external environment (person-in-situation) must be explained. Finally, the sample of the findings is Chinese college students of the general population: the sample did not include those who experienced past trauma. Therefore, future studies should investigate the resilience of different groups to verify the accuracy of this study's results.

## Data availability statement

The original contributions presented in the study are included in the article/supplementary material, further inquiries can be directed to the corresponding author.

## Ethics statement

The studies involving human participants were reviewed and approved by the Human Subjects Ethics Sub-Committee of East China University of Science and Technology. The patients/participants provided their written informed consent to participate in this study.

## Author contributions

RL: conceptualization, data curation, formal analysis, investigation, visualization, and writing-original draft. WD: conceptualization, methodology, project administration, supervision, validation, and writing—review. All authors contributed to the article and approved the submitted version.
